# Correction of Residual Aortic Cusp Prolapse After Resuspension Repair in a Patient With Aortic Regurgitation

**DOI:** 10.1016/j.atssr.2025.08.006

**Published:** 2025-08-29

**Authors:** Yutaka Okita, Ryota Takahashi, Kotaro Tsunemi, Takanori Oka

**Affiliations:** 1 Department of Cardiovascular Surgery, Cardio-Aortic Center, Takatsuki General Hospital, Takatsuki, Japan

## Abstract

A unique technique of correcting a residual prolapse after repairing the congenitally bicuspid valve has been reported. The patient was a 51-year-old woman with a regurgitant bicuspid aortic valve. She underwent a cusp-sparing aortic root replacement with a cusp resuspension technique. After aortic declamping while the heart was beating, a residual cusp prolapse was corrected by pulling out the resuspension suture along the free margin of the aortic cusp under guidance of transesophageal echocardiography.

Aortic valve–sparing root replacement has prevailed because of several advantages over prosthetic valve replacement. However, cusp integrity can be assessed during aortic cross-clamping by using visual, special caliper, or endoscopic examination. Here we report a technique to adjust cusp coaptation while the heart is beating under guidance of intraoperative transesophageal echocardiography (TEE).

A 51-year-old woman was referred to us (Takatsuki General Hospital, Takatsuki, Japan) because of severe aortic regurgitation (AR), although she was asymptomatic. Her body weight was 50 kg, her height was 155 cm, and her body surface area was 1.47 m^2^. She had a congenitally bicuspid valve, consisting of a fused right coronary cusp and noncoronary cusp (NCC) and a nonfused left coronary cusp (LCC). A thick calcified raphe was found at the fused cusp; the sinus of Valsalva was symmetric, and the commissure angle was 180° (Figure 1A). A clear prolapse of the fused cusp was recognized. The left ventricular endodiastolic dimension was 50.3 mm, the endosystolic dimension was 32.4 mm, and the left ventricular ejection fraction was 0.65. The diameter of the basal ring was 24.5 mm, the sinus of Valsalva was 33.9 mm, the sinutubular junction was 29.3 mm, and the ascending aorta was 45 mm. Cusp geometric height was 19 mm in the LCC and 19 mm in the fused cusp. The height from the nadir of the NCC to the top of the NCC-LCC commissure was 20 mm. The diameter of the ascending aorta was 45 mm, and the aortic arch diameter was 42 mm.

**Figure 1:**
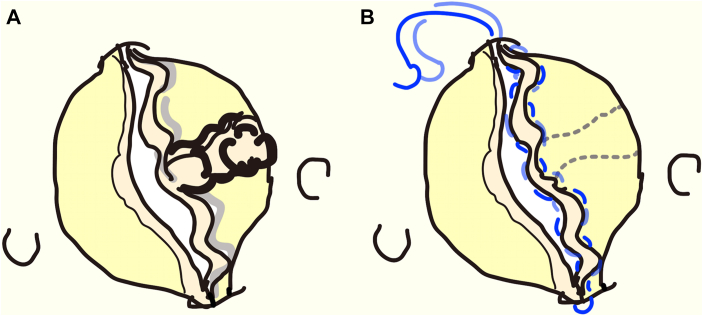
( A) Bicuspid aortic valve with a fused right coronary cusp and noncoronary cusp. A thick, calcified raphe was found in the fused cusp. (B) Cusp resuspension technique in the fused cusp (right coronary cusp and noncoronary cusp) by using CV-7 expanded polytetrafluoroethylene (Gore-Tex W.L. Gore & Associates) sutures (blue). Two parallel CV-7 sutures were placed along the free margin.

A short type of 26-mm Valsalva graft, the diameter of 26 mm and the height of the graft sinus of 22 mm, was used for the reimplantation technique of the aortic root. The calcium plaque at the fused cusp was carefully removed, and additional resuspension sutures, 2 parallel mattress sutures of CV-7 expanded polytetrafluoroethylene (Gore-Tex, W.L. Gore & Associates) along the free margin, were placed to obtain 10 mm of the effective height (Figure 1B, Figure 2). Coronary buttons were reattached at the side holes of the graft by using 5-0 polypropylene (Nespilene, Alfresa) suture, and distal anastomosis was performed.

**Figure 2:**
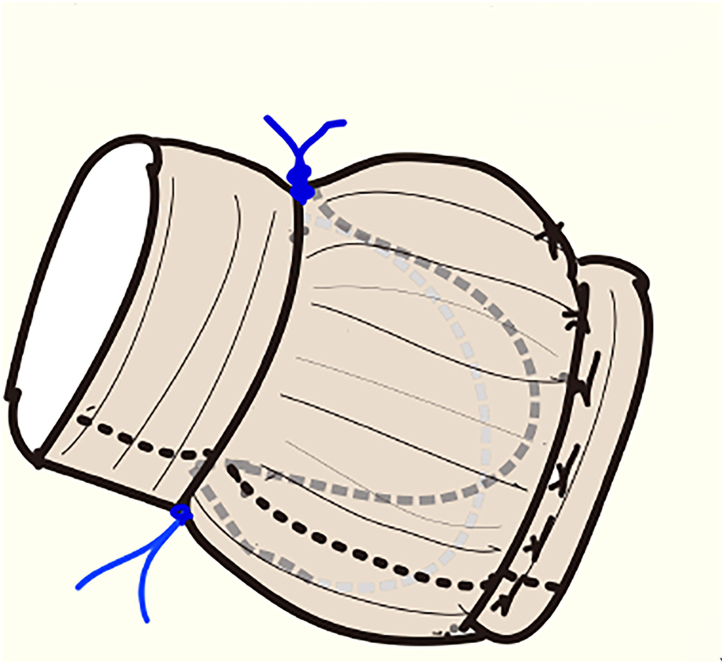
Expanded polytetrafluoroethylene (Gore-Tex, W.L. Gore & Associates) sutures were tied outside of the graft.

At 23 °C of tympanic temperature, circulatory arrest was achieved. Total aortic arch replacement was performed using the antegrade bilateral cerebral perfusion at the zone II aortic arch. Distal circulatory arrest time was 17 minutes, and systemic rewarming was started. After graft-to-graft anastomosis, the aortic clamp was released, and spontaneous cardiac beating was regained. However, TEE showed an eccentric aortic regurgitant jet from the prolapsing fused cusp. While monitoring the TEE, we pulled the tied end of the Gore-Tex sutures out 10 mm and fixed it to the graft wall by using a 5-0 Nespilene suture (Figure 3). The AR disappeared (Video).

**Figure 3:**
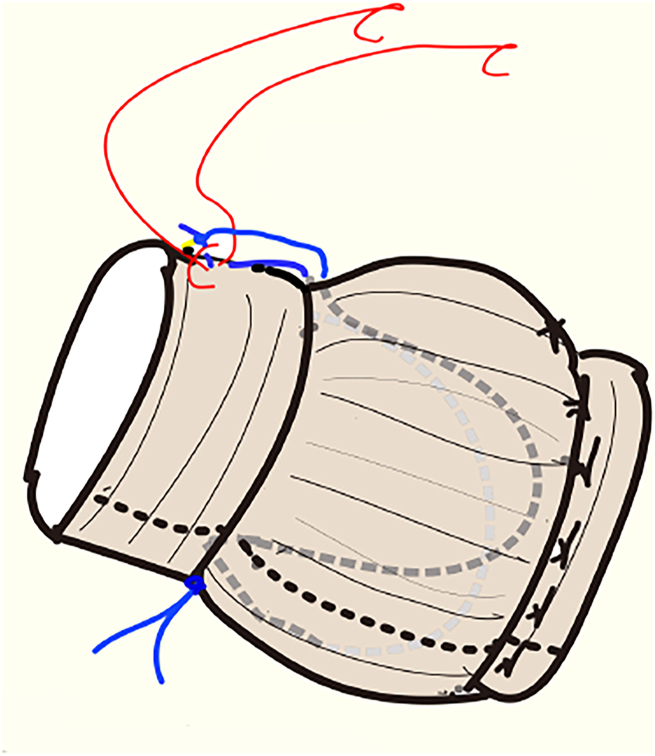
The tied end of the expanded polytetrafluoroethylene (Gore-Tex, W.L. Gore & Associates) sutures was pulled out 10 mm and fixed to the graft wall using 5-0 polypropylene (Nespilene, Alfresa) suture (red).

We have applied the same technique in 5 patients, and their mean age was 57.2 (SD 7.3) years, median age 60 years (range, 48-65 years). All patients had severe AR preoperatively, 2 had bicuspid aortic valves, and 3 had tricuspid cusps. Intraoperative TEE showed the disappearance of the eccentric jet at the aortic valve. Follow-up ranged from 0.2 to 51 months, with a mean follow-up of 27.6 (23.8) months and a median of 34 months. The latest echocardiograms showed degrees of AR in these patients: none in 1 patient, mild in 3, and mild to moderate in 1. No patients had left ventricular dilatation greater than 50 mm in end-diastole.

This study was approved by the Internal Review Board of the Takatsuski General Hospital: (#2022-47) on February 28, 2023.

## Comment

Valve-sparing aortic root replacement has been widely applied in patients with annuloaortic ectasia and AR.^1^ The additional aortic cusp repair has been also tried by aggressive surgeons in patients who had organic cusp lesions.^2^, ^3^, ^4^

Intraoperative evaluation of the competency of the aortic cusp after repair should include visually testing, measurement of the effective height using the Schafer calipers,^5^ root pressure measurement by injecting the solution before coronary anastomosis,^6^ and direct endoscopy.^7^ However, these methods have limitations in observation under static conditions. TEE is an effective tool for assessing cardiac valve function after resumption of the beating heart.

The resuspension technique for repairing the aortic cusps by using CV-7 Gore-Tex sutures^2^ has several advantages over central plication of the Arantius nodules, such as evenly distributed mechanical stress along the entire length in the free margin of the aortic cusp or a proportional shortening of the cusp free margin achieved even in the presence of thickened, rigid tissue. Another advantage of the resuspension technique should be a possible readjustment of the cusp coaptation after a cardiac beating while monitoring the images by TEE.

In conclusion, we present a technique of correcting aortic cusp prolapse while the patient’s heart is beating.
